# Supplementary material for: Lung NR3C1+ and CXCR6high T cells distinguish immunopathogenesis of human emphysema
Source: Commun Biol. 2025 Sep 24;8:1353. doi: 10.1038/s42003-025-08698-1 (PMC12460644; doi:10.1038/s42003-025-08698-1)
Supplement: Supplementary file 2 — Supplemental Material [file 42003_2025_8698_MOESM2_ESM.pdf]

Supplementary Materials

Lung NR3C1$^+$ and CXCR6^{high} T cells distinguish immunopathogenesis of human emphysema

Yun Zhang[1,2], Maor Sauler[3], David B. Corry[1,2,4,5], Scott A. Ochsner[6], Sarah Perusch[5], Li-Zhen Song[1], Joshua Malo[7], Raul San Jose Estepar[8], *Francesca Polverino[1,2,9], *Farrah Kheradmand[1,2,4,5]

[1]Department of Medicine, Division of Pulmonary, Critical Care Medicine, and Sleep, Baylor College of Medicine, Houston, TX, United States, 77030

[2]Department of Pathology and Immunology, Baylor College of Medicine, Houston, TX, United States, 77030

[3] Department of Medicine, Yale University, New Haven, CT, United States, 06520

[4] Biology of Inflammation Center, Baylor College of Medicine, Houston, TX, United States, 77030

[5] Center for Translational Research on Inflammatory Diseases (CTRID), Michael E. DeBakey Department of Veterans Affairs, Houston, TX, United States 77030

[6]Department of Molecular and Cellular Biology, Baylor College of Medicine, Houston, TX, United States, 77030

[7] Department of Medicine, Division of Pulmonary, Critical Care Medicine, and Sleep, University of Arizona, Tucson, AZ 85721

[8] Division of Radiology, Brigham and Women's Hospital, Harvard Medical School, Boston, MA 02115

[9] Department of Medicine, Asthma and Airway Disease Research Center, University of Arizona, Tucson, AZ 85721

*Corresponding authors:

Farrah Kheradmand, MD, farrahk@bcm.edu

Francesca Polverino, MD, PhD, francesca.polverino@bcm.edu

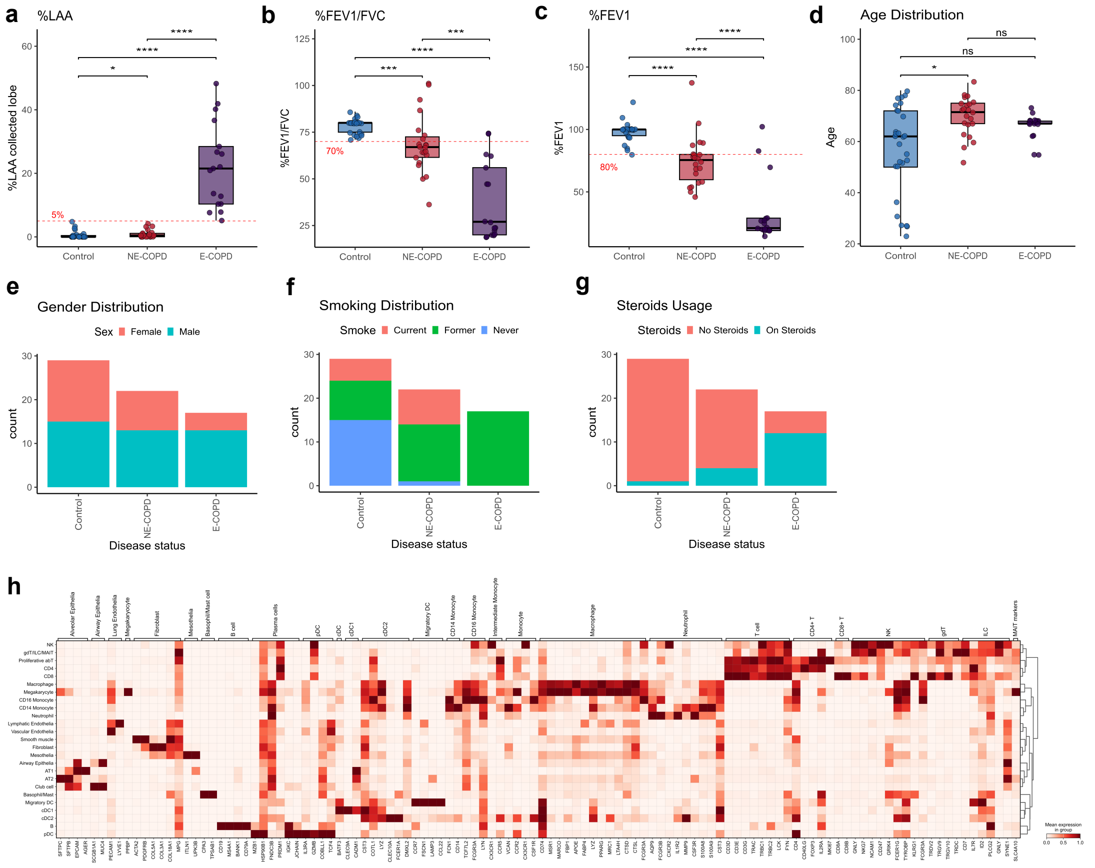

Supplemental Figure 1: Patient characteristics and major lineage markers expression for identified cell types in the in-house discovery dataset. (a-c) Disease metrics include emphysema severity (%LAA) and airflow obstruction (%FEV1/FVC, %FEV1). (d, e, f, g) Patient characteristics include age, gender distribution, history of smoking, and steroid therapy status. Statistical significance between the three groups was determined using the Kruskal-Wallis's test. Pairwise comparisons were performed using the Wilcoxon rank-sum test with Holm's correction. Significance code: p<0.05(*), p<0.01(**), p<0.001 (***). (h) Heatmap of major cell type marker gene expression in identified cell types in the in-house discovery dataset. LAA: Low Attenuation Area; FEV1: Forced expiratory volume in 1 second; FVC: Forced vital capacity.

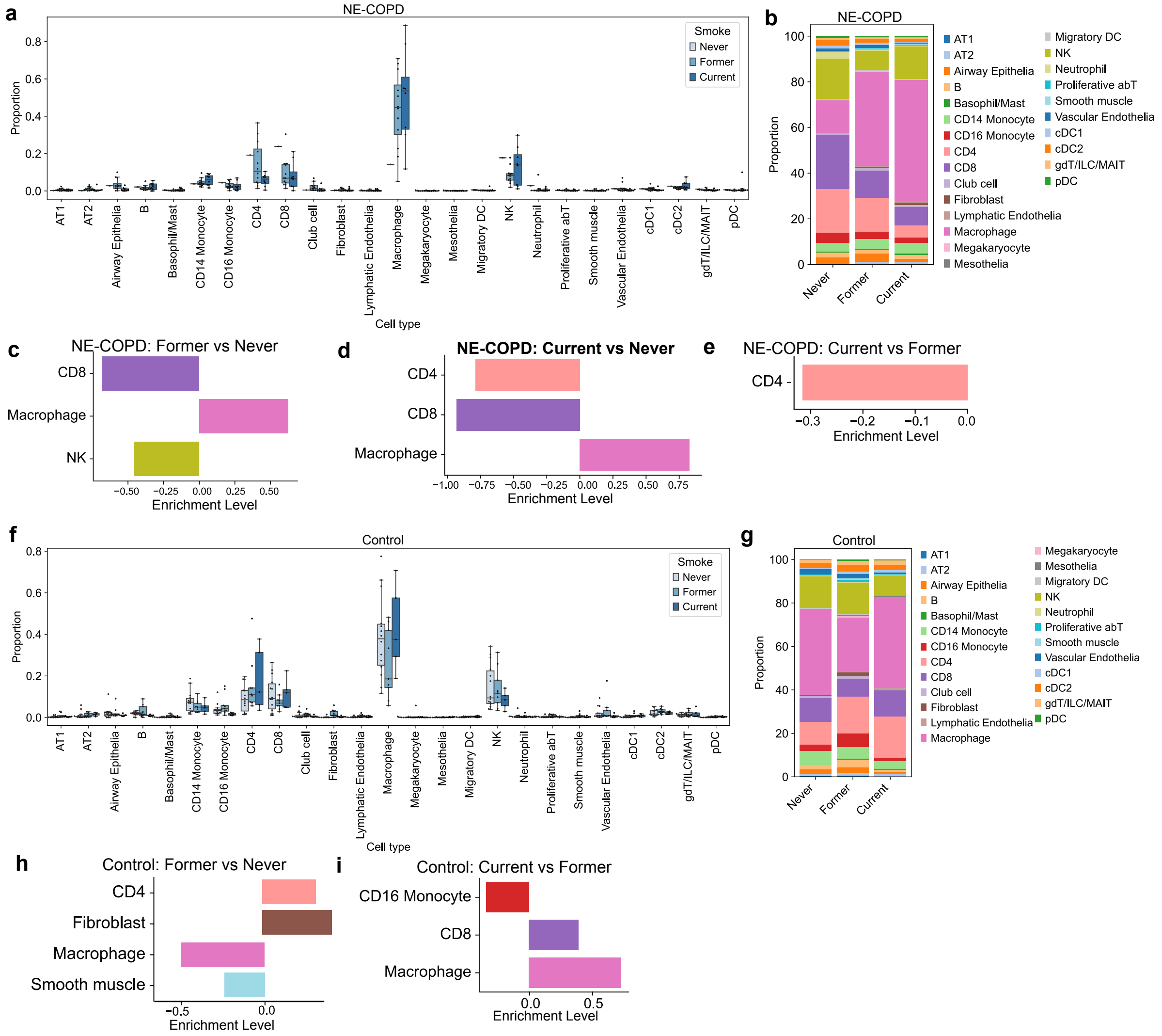

Supplemental Figure 2: Effects of smoking in NE-COPD and Control. Effects of smoking were assessed using scCODA for NE-COPD (a-e), and control groups (f-i). Proportions of cell types of individual patients in (a) NE-COPD and (f) Control. Collective proportions for each disease group in (b) NE-COPD and (g) Control. Enrichment levels of significantly enriched cell types between former and never smokers in (c)NE-COPD and (h) Control. (d) Enrichment levels of significantly enriched cell types between Current and Never smokers in NE-COPD. Enrichment levels of significantly enriched cell types between Current and Former smokers in (e) NE-COPD and (i) Control. The false discovery rate threshold for scCODA enrichment analyses was set at 0.25.

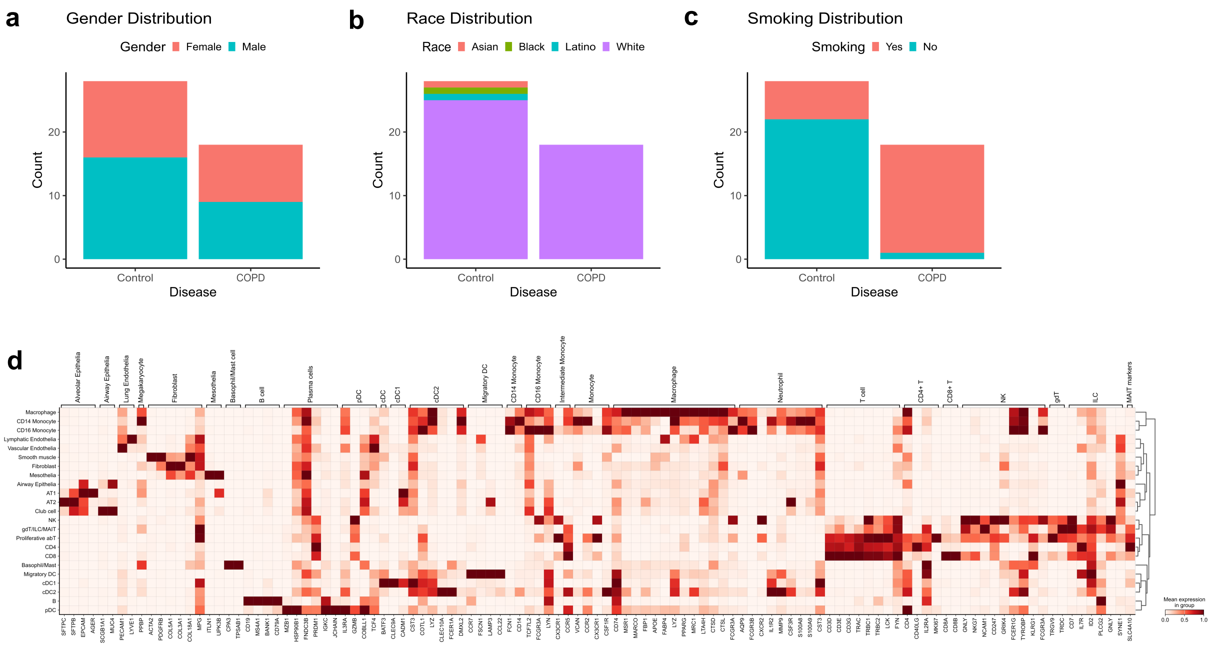

Supplemental Figure 3: Patient characteristics and major lineage markers expression for identified cell types in the published validation dataset. (a-c) Barplots of patient characteristics data including gender distribution, race distribution, and history of smoking in control and End-stage COPD cohorts in the published validation dataset. (d) Heatmap of major cell type marker gene expression in identified cell types in the published validation dataset.

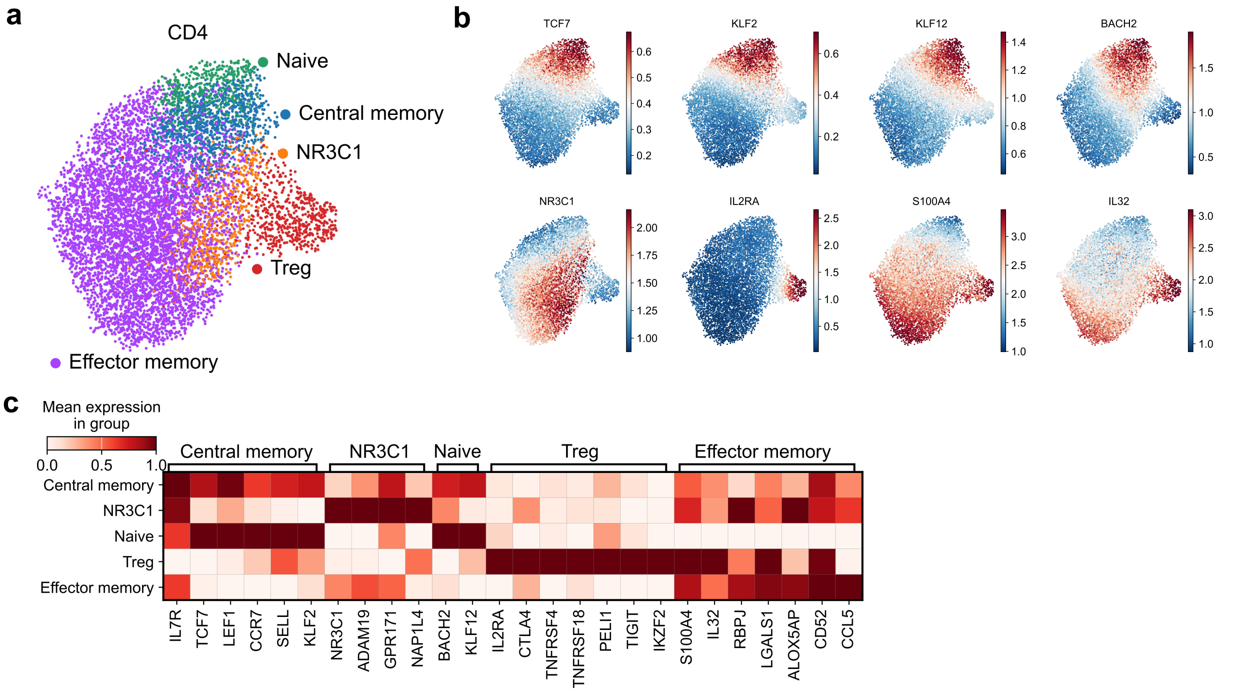

Supplemental Figure 4: Identified CD4 subsets in the published validation dataset. (a) UMAP embedding of CD4 subsets. (b) UMAP embedding of major markers for CD4 subsets in the published validation dataset: Central memory (TCF7, KLF2), Naïve (KLF12, BACH2), NR3C1 CD4 (NR3C), Treg (IL2RA), Effector memory (S100A4 and IL32). (c) Heatmap of gene signature expression of identified CD4 subsets in the validation dataset.

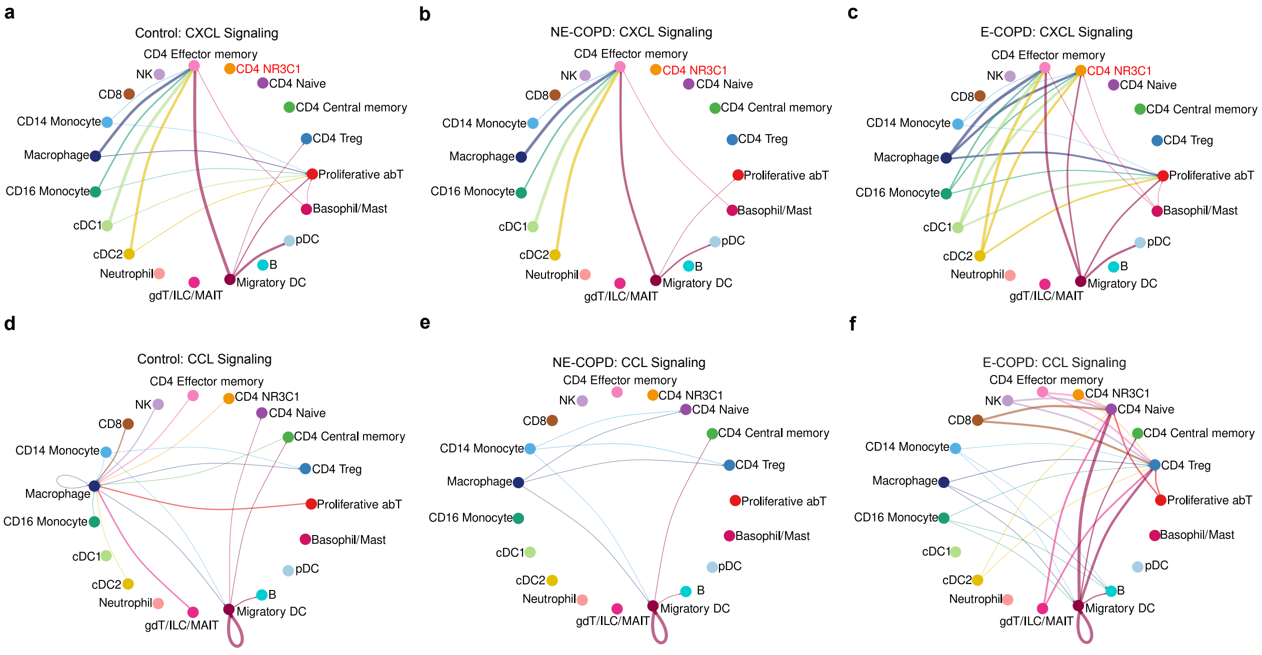

Supplemental Figure 5: Global interaction patterns between cell types. CXCL (a-c) and CCL signaling (d-f) were estimated using CellChat. The width of the chord represents the strength of the signals between connected cell types. Looping chords indicate autocrine interactions.

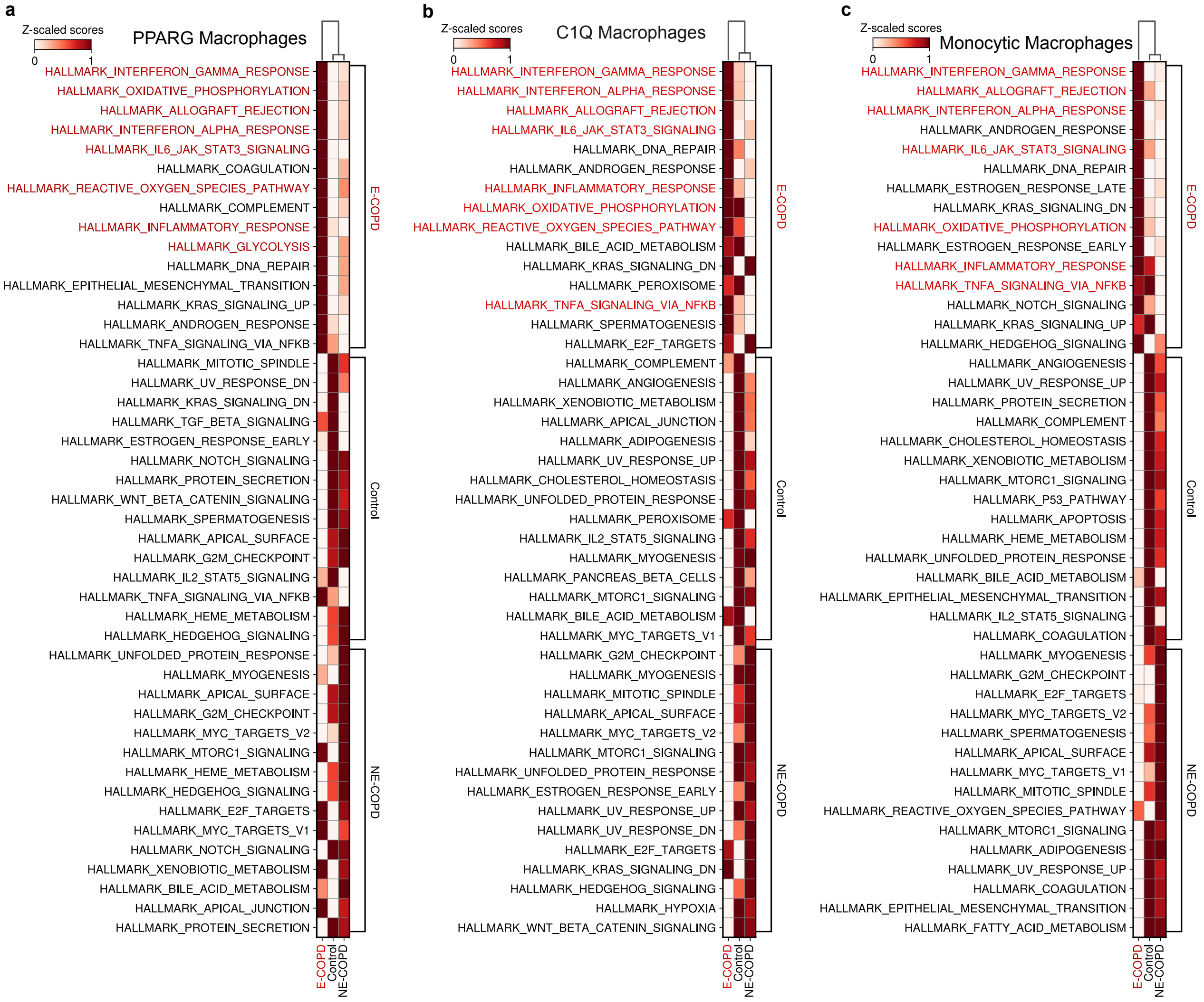

Supplemental Figure 6: Increased inflammatory pathways activities in pulmonary macrophages in E-COPD. Functional enrichment of biological terms with overrepresentation analysis (ORA) using decoupleR in PPARG ITGB8 macrophage (a), C1Q macrophages (b), and Monocytic macrophages (c) across 3 disease groups. Hallmark gene sets were used for mapping pathway activity.

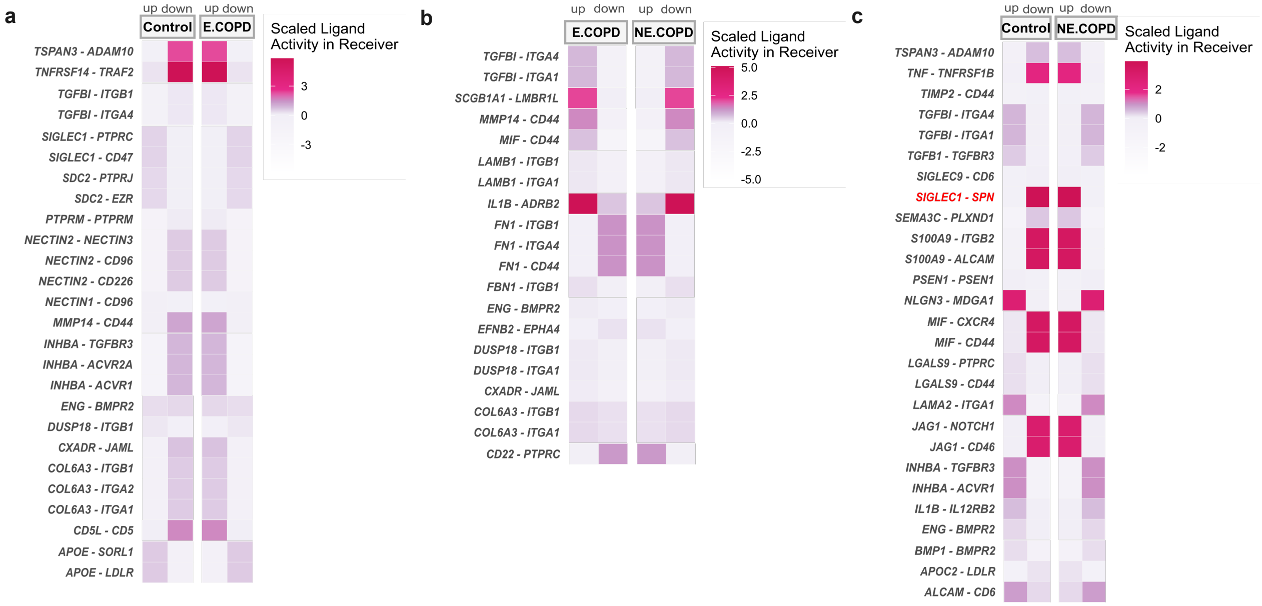

Supplemental Figure 7: Pairwise comparisons of differential receptor-ligand pairs between PPARG macrophages (Sender) and NR3C1 CD4 T cells (Receiver). Pairwise comparison result of differentially regulated ligand-receptor pairs between PPARG ITGB8 macrophages and NR3C1 CD4 T cells. (a) E-COPD vs Control, (b) E-COPD vs NE-COPD, (c) NE-COPD vs Control

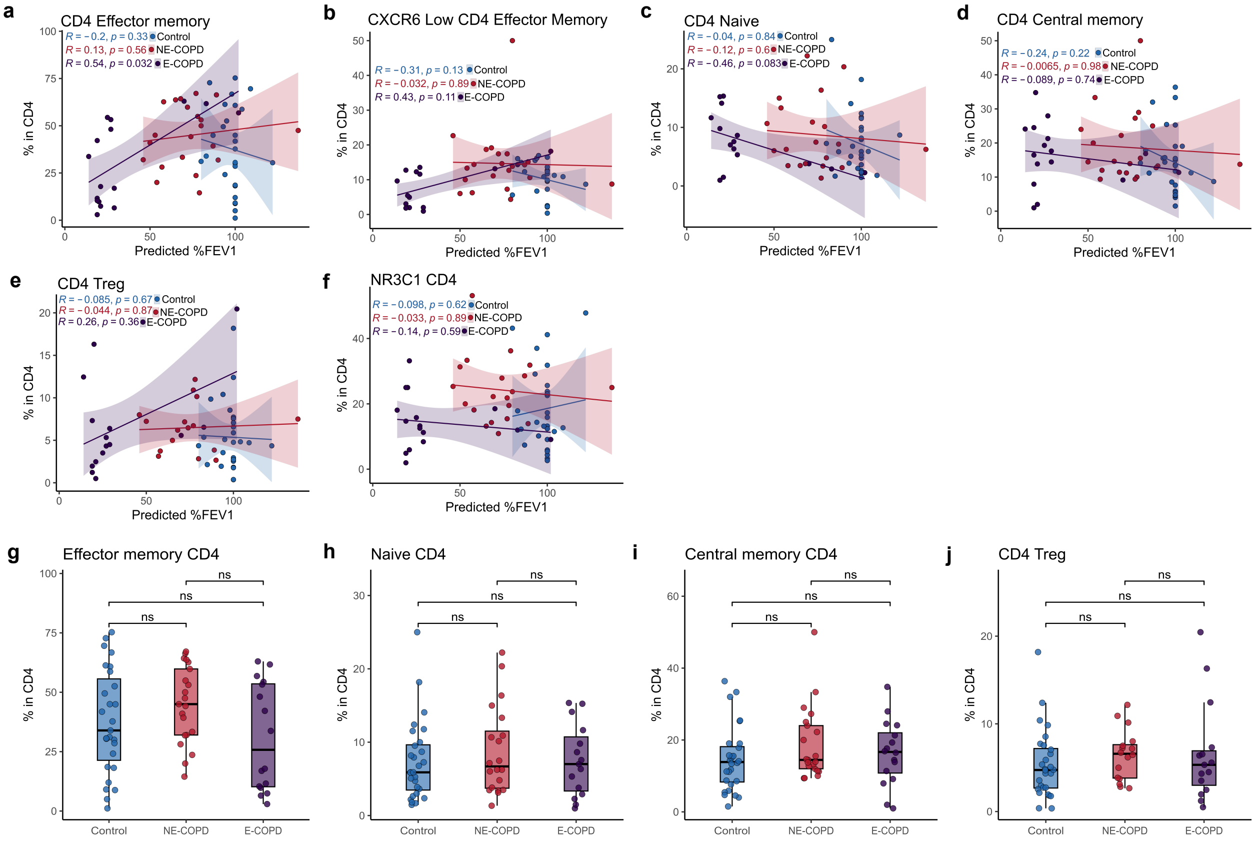

Supplemental Figure 8: Correlation of CD4 subsets with lung function and relative abundances of CD4 subsets across disease groups. Spearman correlation of the percentages of (a) CD4 effector memory, (b) CXCR6Low CD4 effector memory, (c) CD4 naive T cells, (d) CD4 central memory, (e) CD Treg and (f) NR3C1 CD4 T cells in CD4 T cells and %FEV1 in three disease groups. Proportions of (g) effector memory CD4, (h) naive CD4, (i) central memory CD4 and (j) CD4 Treg in CD4 T cells across all three disease groups.

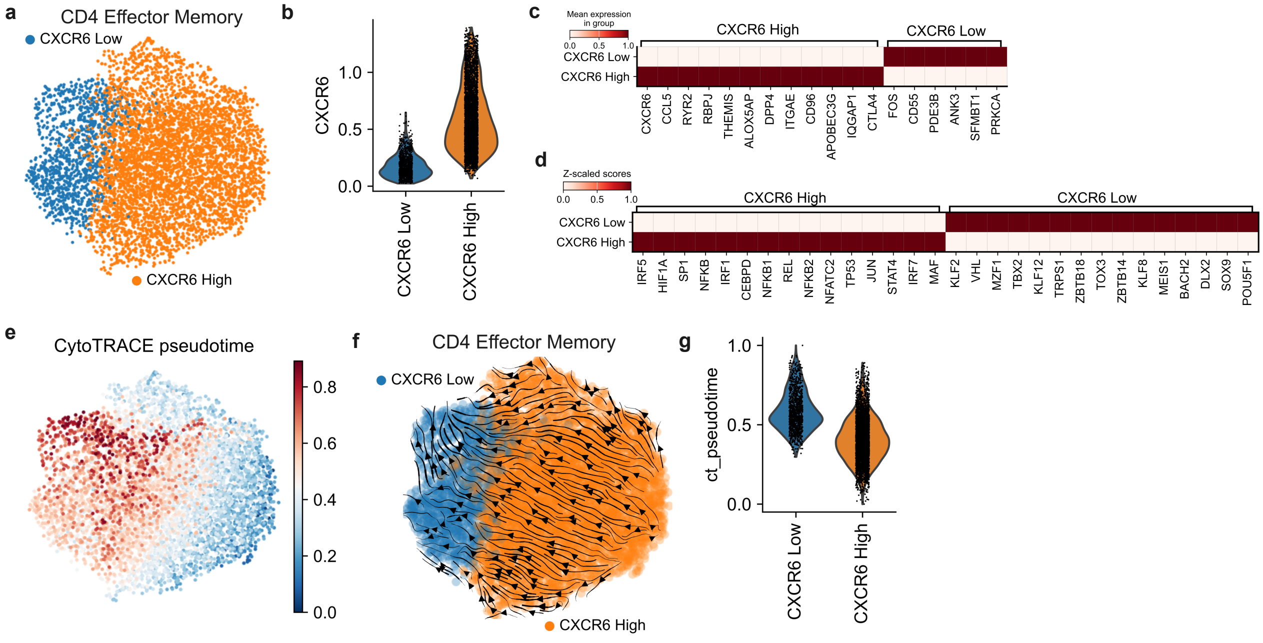

Supplemental Figure 9: CXCR6-based classification of CD4 effector memory in the published validation dataset. (a) UMAP embedding of identified CD4 effector memory subsets. (b) Violin plot of CXCR6 expression in CD4 effector memory subsets in the validation dataset (c) Heatmap of gene signature expression of identified CD4 effector memory subsets in the validation dataset. (d) Heatmap of transcription factor activities of CD4 effector memory subsets in the validation dataset. (e) UMAP embedding of CytoTRACE pseudotime in CD4 effector memory subsets. (f) CellRank estimation of CD4 effector memory subsets differentiation trajectory. (g) Violin plot of CytoTRACE pseudotime in CD4 effector memory subsets.

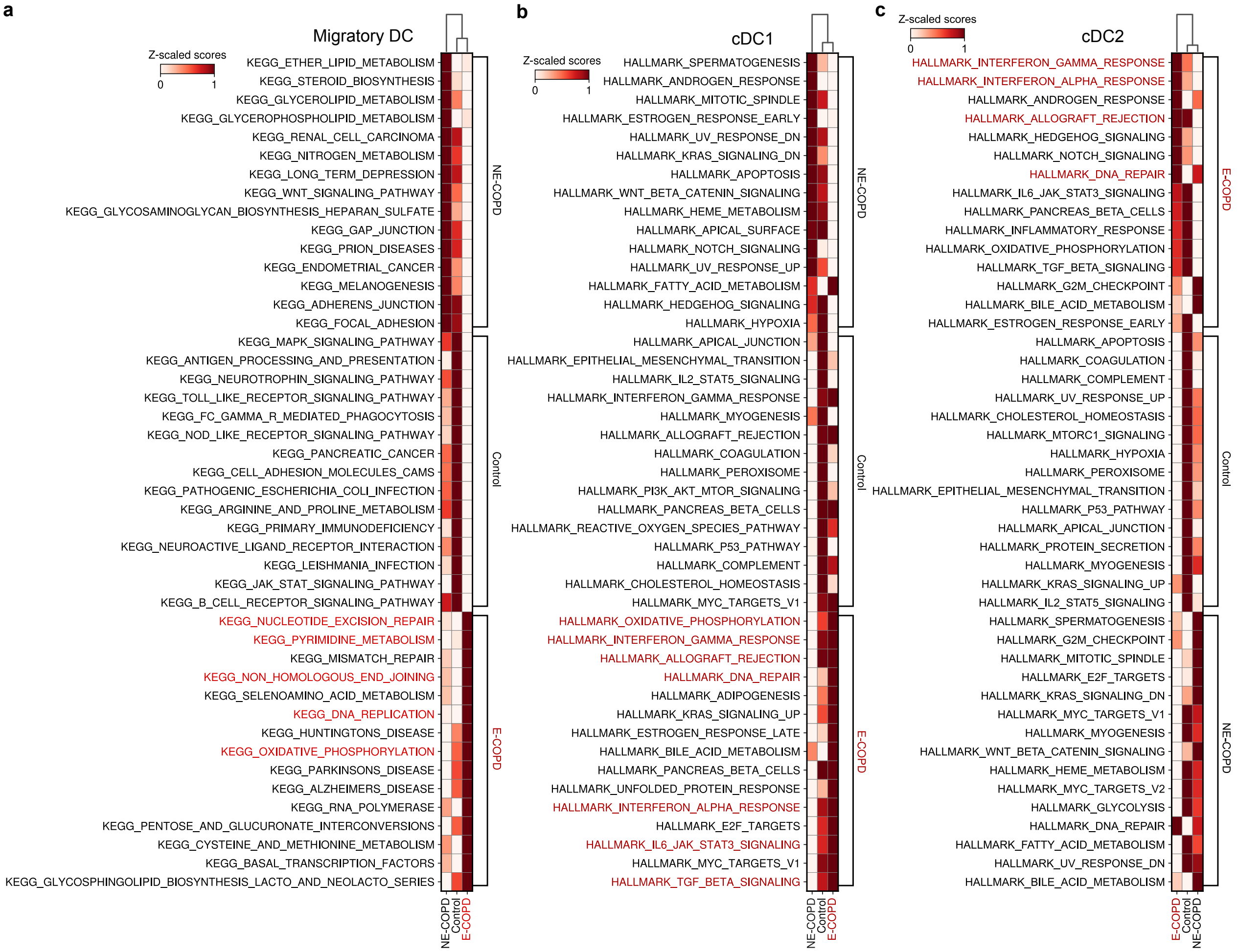

Supplemental Figure 10: Dendritic cells in E-COPD upregulate oxidative phosphorylation and DNA damage responses. Functional enrichment of biological terms with overrepresentation analysis using decoupleR in Migratory DC (a), cDC1 (b), and cDC2 (c) across three groups in the in-house discovery dataset. Hallmark, KEGG, and Gene Ontology Biological processes were used for mapping pathway activity.
